# Comparative efficacy and safety of antivascular endothelial growth factors for central retinal vein occlusion: A protocol for systematic review and network meta-analysis

**DOI:** 10.1097/MD.0000000000028283

**Published:** 2021-12-30

**Authors:** Zhigao Liu, Shuya Wang, Aihua Ma, Bojun Zhao

**Affiliations:** a First Clinical Medical college, Shandong University of Traditional Chinese Medicine, Jinan, Shandong, China; b Department of Pediatrics, Shandong Provincial Hospital Affiliated to Shandong First Medical University, Jinan, Shandong, China; c Department of Ophthalmology, Shandong Provincial Hospital Affiliated to Shandong First Medical University, Jinan, Shandong, China.

**Keywords:** central retinal vein occlusion, macular edema, network meta-analysis, protocol, vascular endothelial growth factor

## Abstract

**Background:**

Central retinal vein occlusion (CRVO) is one of the most common retinal vascular diseases, which is closely related to systemic diseases like hypertension, diabetes and arteriosclerosis. Due of its blinding, it will seriously reduce the quality of life. Macular edema (ME) caused by CRVO is one of the serious complications of visual impairment. We found that the severity of ME in CRVO was positively associated with vascular endothelial growth factor (VEGF) in the anterior chamber. With the accelerated pace of modern life and the changed dietary structure, the incidence of this disease will continue to rise. Therefore, it is of great practical significance to seek effective treatment methods. Intraocular injection of anti-VEGF can effectively alleviate ME and improve visual acuity, showing excellent clinical application prospects. In recent years, there have been some new understandings and advances on the etiology and treatment methods of the present disease, such as the deepening into the molecular biology and gene level. Clinical studies on the efficacy of the disease have emerging. Therefore, a network meta-analysis (NMA) of anti-VEGF treatment for CRVO is particularly necessary to systematically compare its efficacy.

**Methods:**

The two reviewers will comprehensively retrieved electronic databases such as PubMed, The Cochrane Library, Wanfang database, Web of Science, Chinese Scientifific Journals Database, EMBASE, China National Knowledge Infrastructure, and China BioMedical Literature. A randomized controlled trial for CRVO against VEGF between January 2010 and June 2021 was included according to the relevant content of the study. In addition, 2 researchers will screen the literature to assess the risk bias for the included articles. We will evaluate the collected evidence and data using a Bayesian NMA method, and analyzed it with STATA and WinBUGS software.

**Results:**

Anti-VEGF is one of the effective methods for ME in CRVO patients, accordingly, this study will evaluate its efficacy and safety using a Bayesian NMA system.

**Conclusion:**

This study can provide an effective rationale for the clinical application of anti-VEGF for CRVO, contribute to the treatment of CRVO and patient condition rehabilitation in clinical work.

**Ethics and dissemination:**

Do not require.

**INPLASY registration number:**

INPLASY2021110073.

## 1 Introduction

Central retinal vein occlusion (CRVO), also known as retinal apoplexy, is one of the common clinical retinal vascular diseases.^[1]^ Its typical clinical manifestation is sudden painless monocular vision loss.^[2]^ In 1878, von Michel believed that the cause of the disease was venous embolism. At present, CRVO has become one of the main causes of blindness,^[3]^ which negatively affects the quality of life, increases the psychological burden and financial burden.[^4^,^5^]

Retinal vein occlusion is a common retinal vascular fundus disease in clinic and a second blinding fundus disease after diabetic retinopathy. The common vessel of retinal vein occlusion is CRVO. A study shows that there are about 2.5 million CRVO patients worldwide.^[6]^ Persistent macular edema (ME) is the most important cause of visual impairment. According to the data, the incidence of ME in CRVO is 46.7%, and the low visual acuity is 57.4%.^[7]^

At present, there are still many controversies on the classification, etiology, and treatment of CRVO, because of the severity of ME caused by CRVO. But some results have also been achieved. Studies on its etiology have shown that the severity of ME in CRVO is closely associated with vascular endothelial growth factor (VEGF) in the anterior chamber and positively with ME severity. Among these, VEGF levels were higher in the vitreous and anterior chambers than the nonischemic CRVO, ischemic CRVO. Expression of VEGF facilitates the clinical assessment of macular oedema severity in CRVO patients. Anti-VEGFs drugs are the primary means of treatment, which have a significant effect in solving ME and improving vision.^[8]^ Intravitreal bevacizumab can effectively treat retinal vein occlusion and CRVO in the short term. Anti-VEGF is a powerful weapon of relieve ME and improve vision.^[9]^

Neovascularization is a common pathological change of many common eye diseases, and VEGF is an important promoter in the process of neovascularization. In recent years, intravitreal injection of anti-VEGF drugs has opened up a new direction for the treatment of neovascular eye diseases, and achieved good results. It has been verified in numerous systemic reviews and clinical trials that, anti-VEGF is the main method in the treatment of CRVO. However, its safety and effectiveness have not been compared. Network meta-analysis can take full advantage of clinical data and compare the efficacy of more than 3 treatments, with greater superiority over traditional meta-analyses. Therefore, we will use it to compare the safety and efficacy of anti-VEGF treatment of CRVO.

## 2 Materials and methods

### 2.1 Study registration

We will follows the PRISMA-P guidelines, and register on the international platform of registered systematic review and meta-analysis protocols. The registration number is INPLASY2021110073 (URL = https://inplasy.com/inplasy-2021-11-0073/).

### 2.2 Inclusion criteria

#### 2.2.1 Type of study

We will collect all eligible randomized controlled trials of anti-VEGF for CRVO, together with relevant clinical trials. We collected only articles published in Chinese or English.

#### 2.2.2 Participants

All the patients with CRVO were enrolled. The diagnosis of CRVO will follow the guidelines for CRVO.

#### 2.2.3 Interventions

In the present study, patients in experimental group were given anti-VEGF treatments. The control group was treated with other therapies other than anti-VEGF.

#### 2.2.4 Outcomes

The outcomes of our interest are the change of the best-corrected vision, central retinal thickness,[^10^,^11^] the proportion of patients with vision change of 15 or more ETDRS characters, the proportion of patients with vision better than 20/40 Snellen or worse than 20/200 Snellen, the regression rate of ME, the proportion of patients with neovascular complications, and the change of NEI VFQ-25 score before and after treatment.

### 2.3 Database and search strategy

We retrieved a large number of electronic databases using different search strategies, such as PubMed, The Cochrane Library, Wanfang database, Web of Science, Chinese Scientifific Journals Database, EMBASE, China National Knowledge Infrastructure, and China BioMedical Literature. During retrieval, a combination of medical subject titles and free text terms was used, include “Retinal Vein Occlusions, Retinal Vein Thromboses, CRVO, Branch Vein Occlusion”. The PubMed search strategy are shown in Table 1.

**Table 1:** Detailed search strategy for PubMed.

NO.	Search item
1#	“Central Retinal Vein Occlusion” [MeSH Terms]
2#	“Retinal Vein Occlusions” [Title/Abstract] or “Retinal Vein Thrombosis” [Title/Abstract] or “•Retinal Vein Thromboses” [Title/Abstract] or “•Central Retinal Vein Occlusion” [Title/Abstract] or “•Branch Vein Occlusion” [Title/Abstract] or “•Branch Vein Occlusions” [Title/Abstract] or “•Retinal Branch Vein Occlusion” [Title/Abstract]
3#	1# or 2#
4#	“Anti-Vascular Endothelial Growth Factor” [MeSH Terms]
5#	“•DC101 Mab” [Title/Abstract] or “monoclonal antibody DC101” [Title/Abstract] or “HuMV833” [Title/Abstract] or “HuMV833 monoclonal antibody” [Title/Abstract] or “Mvasi” [Title/Abstract] or “Bevacizumab-awwb” [Title/Abstract] or “Bevacizumab awwb” [Title/Abstract] or “Avastin” [Title/Abstract]
6#	4# or 5#
7#	“randomized controlled trial” [Title/Abstract] or “controlled clinical trial” [Title/Abstract] or “Randomized” [Title/Abstract] or “random allocation” [Title/Abstract] or “Randomly” [Title/Abstract]
8#	3# and 6# and 7#

### 2.4 Study selection and data extraction

Two researchers independently conducted literature screening and data extraction based on the inclusion and exclusion criteria of the study. All the data were extracted directly from the literature and verified repeatedly to ensure the accuracy of the data. The records included efficacy data and safety data. For controversial data, consult with experienced clinicians and statistical experts to reach a consensus.

If the literature does not provide complete data, try to communicate with the original author to obtain the missing data. If the original author's reply is not received after 4 weeks, other feasible and accepted methods are used to estimate the absent data, such as replacing the missing standard deviation with the combined standard deviation of other studies.

Efficacy data:

(1) The best-corrected visual acuity changes at 1, 6, and 12 months, represented by ETDRS characters.
(2) The central retinal thickness changes at 1, 6, and 12 months, represented by μm.
(3) Best-corrected visual acuity improved or deteriorated by 15 or more ETDRS characters (equivalent to 3 lines of Snellen chart) at 6 months.
(4) At 6 months, the cases of best corrected visual acuity was ≥20/40 or ≤20/200 Snellen.
(5) The number of cases with ME in 6 months.
(6) The number of cases with neovascular complications in 6 months.
(7) The change of Nei VFQ-25 in 6 months.

Safety data: the number of cases with intraocular or systemic adverse events, and counted by disease type.

### 2.5 Risk of bias assessment

The risk of literature bias was assessed using the Cochrane collaborative network quality evaluation tool, which is generated from the random number series, distributed and hidden, and whether the blind method is adopted or not. The results were divided into high-risk bias, low-risk bias, and unknown risk bias. After the evaluation, 2 researchers conducted the study Crosscheck.

### 2.6 Statistical analysis

Revman 5.2.3 software was used for statistical analysis and processing of the data, and the entered data was checked repeatedly before the analysis to ensure accuracy. The effect model used in the analysis depends on the heterogeneity among the studies. When *P* ≤ .05, the difference between the 2 groups was statistically significant. The analysis results are expressed in the form of a forest map.

Measurement data or continuous value: The results were expressed as mean value ± mean difference and 95% confidence interval between the 2 groups.

Count data or dichotomous data: it is expressed numerically, and the combined risk ratio is used as the effect scale. The analysis results, risk ratio and 95% confidence interval are expressed.

### 2.7 Assessment of heterogeneity

The heterogeneity among the studies was evaluated by I^2^. Heterogeneity test ≥ 50%, *P* ≤ .1, indicating significant heterogeneity between studies. The random effect model was used when I ≤ 50%, *P* ≥ .01, indicating no significant heterogeneity, Fixed effect model was used for analysis.

### 2.8 Subgroup analysis and sensitivity analysis

We will consider the grouping analysis with sufficient data. Because some clinical trials do not provide complete standard deviation, the reliability of analysis results may be reduced by using the standard deviation of other clinical studies instead. Secondly, there are excellent clinical differences between ischemic and nonischemic CRVO in the course of disease and prognosis. Some clinical trials exclude or contain very few patients with ischemic CRVO, which may affect the overall analysis results. Therefore, the following 2 screening conditions were used for sensitivity analysis:

(1) Clinical trials without standard deviation were excluded.
(2) Clinical trials excluding patients with ischemic CRVO, <5% of patients with ischemic CRVO and an unknown proportion of patients with ischemic CRVO were excluded.

### 2.9 Evaluation of publication bias

We will establish a coordinate system with the change of the best-corrected vision, central retinal thickness, the proportion of patients with vision change of 15 or more ETDRS characters, the proportion of patients with vision better than 20/40 Snellen or worse than 20/200 Snellen, the regression rate of ME, the proportion of patients with neovascular complications, and the change of NEI VFQ-25 score as indicators, and draw an inverted funnel diagram in it. Determine whether the raw materials have a publication bias according to whether the inverted funnel map is symmetrical.

### 2.10 Grading the quality of evidence

The grading of recommendations assessment, development and evaluation framework[^12^,^13^] will be used to evaluate the quality of evidence-based medicine.

Evaluation parameters:

(1) Best-corrected visual acuity.
(2) Central retinal thickness.
(3) Number of cases with best-corrected visual acuity not less than 15 ETDRS characters.
(4) The number of cases with neovascular complications.
(5) NEI VFQ-25.

## 3 Discussion

CRVO is one of the most common retinopathy. Tortuous dilated retinal vein and retinal haemorrhage are the main ocular manifestations of retinal vein occlusion It's a record. Visual results are closely related to the ischemic state and integrity of capillaries around fovea. ME and neovascularization are the causes of severe visual loss, and the main complication of the loss of blood pressure. The pathogenesis is still unclear. However, the retinal vessels are anatomically narrowed, especially at the arteriovenous junction, at the cribriform plate The crowding effect of the layer, and many intravascular lesions that cause thrombosis Inflammatory diseases of trauma may lead to vascular occlusion.[^14^,^15^] Retina VEGF and inflammatory cytokines induced by ischemia increase vascular permeability and leakage. The treatment of ME includes macular laser photocoagulation after an operation, anti-VEGF drugs, pstA and dexamethasone were injected into vitreous vitrectomy.[^16^,^17^]

Anti-VEGF drugs have been shown to improve visual and anatomical results significantly. After retinal vein occlusion, the expression level of VEGF increased significantly, and enhanced the exudation of the vascular wall. The possible mechanism^[18]^ is After the formation of retinal vein occlusion, extensive retinal haemorrhage makes the retina absent. Due to the increase of VEGF, the retinal vascular permeability increases, which leads to ME. Anti-VEGF drugs can reduce the exudation of the vascular wall and inhibit the proliferation of vascular endothelial cells.

CRVO is a common retinal vascular disease. ME caused by it is one of the serious complications of visual impairment. Intraocular injection of anti-VEGF can effectively alleviate ME and improve patients’ visual acuity, showing a great clinical application prospect. However, there is still a lack of sufficient evidence-based medical evidence in terms of safety and efficacy. Therefore, we will use network meta-analysis to compare the efficacy of anti-VEGF drugs for CRVO, so as to provide a credible theoretical basis for the clinical application of anti-VEGF drugs for CRVO.

## Author contributions

**Conceptualization:** Zhigao Liu.

**Data curation:** Zhigao Liu, Shuya Wang.

**Formal analysis:** Zhigao Liu, Aihua Ma, Bojun Zhao.

**Funding acquisition:** Shuya Wang.

**Investigation:** Zhigao Liu.

**Methodology:** Zhigao Liu, Aihua Ma, Bojun Zhao.

**Project administration:** Zhigao Liu.

**Resources:** Zhigao Liu.

**Software:** Zhigao Liu, Aihua Ma, Bojun Zhao.

**Supervision:** Zhigao Liu.

**Validation:** Zhigao Liu.

**Visualization:** Zhigao Liu.

**Writing – original draft:** Zhigao Liu, Shuya Wang.

**Writing – review & editing:** Zhigao Liu, Shuya Wang.

## References

[1] Inagaki M Hirano Y Yasuda Y. Twenty-four month results of intravitreal ranibizumab for macular edema after branch retinal vein occlusion: visual outcomes and resolution of macular edema. Semin Ophthalmol 2021;36:482–9. 33617388 10.1080/08820538.2021.1890147

[2] Sarpangala S George NM Kamath YS Kulkarni C. Central retinal vein occlusion secondary to varicella zoster retinal vasculitis in an immunocompetent individual during the COVID-19 pandemic – a case report. Indian J Ophthalmol 2021;69:2532–5. 34427261 10.4103/ijo.IJO_1644_21 PMC8544091

[3] Lulu C. Choroidal thickening in retinal vein occlusion patients with serous retinal detachment. Graefe Arch Clin Exp Ophthalmol 2021;259:883–9. 10.1007/s00417-020-04983-3 33205242

[4] Aya M. Central retinal arterial and venous occlusion in a patient with systemic sclerosis and a review of the related literature. Am J Int Med 2021;9: doi: 10.11648/j.ajim.20210904.19.

[5] Jean-François K. Efficacy and safety of intravitreal aflibercept treat-and-extend for macular edema in central retinal vein occlusion: the CENTERA study. Am J Ophthalmol 2021;227:106–15. 33556381 10.1016/j.ajo.2021.01.027

[6] Bjeloš M. Central retinal artery and vein occlusion as a complication of persistent hyaloid artery – a case report. BMC Ophthalmol 2020;20:434–1434. 33143669 10.1186/s12886-020-01702-8 PMC7607849

[7] Chakraborty D Saha A Pradhan A. A curious case of macular edema in persistent fetal vasculature. Indian J Ophthalmol – Case Rep 2021;1:416–7.

[8] Lee T. Treatment patterns and clinical outcomes for branch retinal vein occlusion: an 8-year experience at a tertiary eye center. J Vitreoretinal Dis 2021;5:412–9. 10.1177/2474126420978874 PMC9976115 37008715

[9] Dewan KS Hentati F Greenlee TE. Age-related differences in presentation and outcomes of anti-VEGF treatment of retinal vein occlusion. Can J Ophthalmol 2021;56:196–204. 10.1016/j.jcjo.2020.09.004 33039322

[10] Tatsumi T. Effects of switching from Anti-VEGF treatment to triamcinolone acetonide in eyes with refractory macular edema associated with diabetic retinopathy or retinal vein occlusion. BioMed Res Int 2020;2020:4529850. 33274211 10.1155/2020/4529850 PMC7695504

[11] Xia JP Wang S Zhang JS. The anti-inflammatory and anti-oxidative effects of conbercept in treatment of macular edema secondary to retinal vein occlusion. Biochem Biophys Res Commun 2019;508:1264–70. 30558792 10.1016/j.bbrc.2018.12.049

[12] Guyatt G Oxman AD Akl EA. GRADE guidelines: 1. Introduction-GRADE evidence profiles and summary of findings tables. J Clin Epidemiol 2011;64:383–94. 21195583 10.1016/j.jclinepi.2010.04.026

[13] Puhan MA Schünemann HJ Murad MH. A GRADE Working Group. Approach for rating the quality of treatment effect estimates from network meta-analysis. BMJ 2014;349:g5630. 25252733 10.1136/bmj.g5630

[14] Teru Asato. Closure of macular hole secondary to ischemic hemi-central retinal vein occlusion by retinal photocoagulation and topical anti-inflammatory treatment. Lasers Med Sci 2021;36:469–71. 32827075 10.1007/s10103-020-03133-9 PMC7881958

[15] D Khokhlova Yu. Optical coherence tomographic patterns in patients with retinal vein occlusion and macular edema treated by ranibizumab: a predictive and personalized approach. EPMA J 2021;12:57–66. 33786090 10.1007/s13167-021-00233-6 PMC7954946

[16] Casselholm de S Lindberg C Epstein D. Neovascular glaucoma in patients with central retinal vein occlusion: A real-life study in the anti-VEGF era. Acta Ophthalmol 2020;99:e7–12. 32548981 10.1111/aos.14500

[17] Gao X. Intravitreal anti-vascular endothelial growth factor injections for macular edema associated with central retinal vein occlusion in patients age 40 years or younger. Ophthalmic Surg Lasers Imaging Retina 2019;50:e96–104. 30998252 10.3928/23258160-20190401-13

[18] Yan Z. YC-1 inhibits VEGF and inflammatory mediators expression on experimental central retinal vein occlusion in rhesus monkey. Curr Eye Res 2018;43:526–33. 29364731 10.1080/02713683.2018.1426102

